# Modification of Hospitalization Risk by Gender and Dementia Status Between the Ages of 85 and 95 in a German Cohort Based on Health Claims Data

**DOI:** 10.1186/s12913-026-14575-2

**Published:** 2026-05-08

**Authors:** Gabriele Doblhammer, Elena Rakuša, Anne Fink

**Affiliations:** 1 German Center for Neurodegenerative Disease (DZNE), Bonn, Germany; 2 Institute for Sociology and Demography, University of Rostock, Rostock, Germany

**Keywords:** Age, Age at death, End of life, Comorbidity, Long-term care need, Nursing home

## Abstract

**Background:**

Hospitalizations among older adults differ by dementia status, gender, and living arrangements. Understanding these differences, particularly in advanced age (age 85 and above), can inform appropriate healthcare strategies.

**Methods:**

Using health claims data for Germany, we followed the 1918 to 1923 birth cohort (*n* = 4,065 men and 13,302 women), who reached age 85 between 2004 and 2009 until death or age 95. Two-level mixed-effects linear probability models with repeated observations were conducted, adjusting for age, gender, dementia status, nursing home residency, dependency on long-term care, comorbidities, and quarter of death.

**Results:**

Men consistently exhibited higher probabilities of hospitalization compared to women, and individuals with dementia (PwD) had a greater probability of hospitalization than those without dementia (non-PwD). Specifically, when compared to male non-PwD, the probability of hospitalization increased by 0.10 (*p* ≤ 0.001) for male PwD; female non-PwD demonstrated a 0.02 (*p* ≤ 0.001) lower probability of hospitalization, while female PwD had an increased probability of 0.06 (*p* ≤ 0.001). Hospitalization probabilities increased with age among non-PwD (men: +0.052 from p_85_=0.14 [95%CI = 0.13–0.14] to p_95_=0.19 [95%CI = 0.18–0.20]), women: +0.021 from p_85_=0.12 [95%CI = 0.12–0.13] to p_95_=0.14 [95%CI = 0.14–0.15]), remained almost stable among male PwD (+ 0.018 from p_85_=0. 24 [95%CI = 0.23–0.26] to p_95_=0.26 [95%CI = 0.24–0.28]), but declined among female PwD (-0.023 from p_85_=0.22 [95%CI = 0.21–0.22] to p_95_=0.20 [95%CI = 0.19–0.20). The quarter of death strongly elevated hospitalization probabilities for all groups, though less so among women with dementia who had a lower probability than women without dementia (-0.04; p_non-PwD_=0.58 [0.58–0.59], p_PwD_=0.54 [0.53–0.55]). Dependence on long-term care significantly reduced hospitalization risk among women, especially those with dementia (-0.5; p_no long-term care_=0.21 [0.21–0.21], p_long-term care_ =0.16 [0.16–0.17]), but showed no substantial effect for men. Nursing home residency increased hospitalization probabilities mainly for women without dementia (+ 0.02), but slightly decreased probabilities for women with dementia (-0.01). Higher comorbidity was consistently associated with greater hospitalization risk.

**Conclusion:**

Gender and dementia status significantly modulate hospitalization risks in advanced age. A gender-sensitive healthcare approach that accounts for dementia status and care needs is crucial for ensuring adequate hospital care in advanced age.

**Supplementary Information:**

The online version contains supplementary material available at 10.1186/s12913-026-14575-2.

## Introduction

In industrialized countries most of population growth is occurring among long-lived individuals at ages 85 and above, who have been described as having lower health-related quality of life, higher levels of functional and cognitive impairment, and an increased use of healthcare services than their younger counterparts [1]. A major cause of their impaired health and poor quality of life is related to dementia, which affects approximately one-third [2] with women at a higher risk [3]. After long-term care, hospitalization is the main driver of health care costs for people with dementia, which are typically twice as high for people with moderate to severe dementia as for people without dementia [4, 5]. The risk of hospitalization is a complex interaction of current age, age at death and proximity to death, modified by gender and the patient’s morbidity profile. People with dementia are hospitalized 1.4 to 4 times more frequently and tend to have longer hospital stays than people without dementia and similar illnesses and are readmitted more often [4, 5]. Dementia is rarely the reason for hospitalization, but it can present large challenges for patients, their caregivers, and healthcare staff when it leads to hospitalization [6]. These challenges can include difficulties with medical and nursing procedures, longer examination and treatment times, and increased staff time due to the patient’s special needs [6]. Dementia patients may also become agitated or aggressive in response to stressors such as fatigue, changes in their environment or caregivers, and excessive demands [7].

Hospitalization can be necessary and beneficial for diagnosing and treating acute illnesses in persons with dementia. However, it may also be associated with unintended adverse consequences, including stress as well as functional and cognitive decline [5]. In some cases, hospitalization for severe illness may accelerate cognitive decline or lead to lasting changes in cognitive functioning that can contribute to the development or progression of dementia [8]. Patients with severe cognitive impairments tend to have higher rates of complications during their hospital stays and longer hospital stays overall, with an average increase of 1.4 days compared to patients with no or moderate cognitive impairments [9]. At the same time, some hospital admissions among older adults may involve conditions that could potentially be managed in outpatient settings [5]. E.g. in Germany, about one-quarter of hospital admissions of cognitively normal and impaired older adults could potentially be treated on an ambulatory basis [9]. This issue becomes particularly relevant near the end of life, when hospitalization rates tend to increase and often peak close to death [10].

Beyond these dementia-specific considerations, hospitalization risks among older adults are also strongly shaped by proximity to death. Hospitalization rates tend to increase substantially near the end of life and often peak shortly before death [10]. Healthcare utilization in the three years before death tends to be lower for women and is modified by cause of death [11], but there is also evidence that for ages 85 and above the cost of hospitalization is higher for females [12]. Defining the end of life in dementia patients, however, is complex due to the potentially long and unpredictable trajectory of dementia. Many persons with dementia may never reach the advanced stages and may die from other causes earlier in the trajectory [13], and only about a quarter of those dying with dementia have severe dementia [5]. Another source of variability is the place of living. While hospitalization rates of German nursing home residents are higher than in other countries [14], most studies have found that persons with dementia living in nursing homes are less likely to be hospitalized at the end of life, which may indicate that they receive less aggressive treatment. However, one study of Germany found no difference in hospitalization rates between residents with and without dementia [10].

Despite growing research on dementia and healthcare utilization, several important gaps remain. First, relatively little is known about hospitalization patterns among long-lived individuals aged 85 and above, even though this age group is expanding rapidly and has the highest prevalence of dementia and mortality. Second, existing studies rarely examine how dementia interacts simultaneously with age, proximity to death, comorbidity, and care context such as long-term care dependency and nursing home residence in shaping hospitalization risks. Third, a gender perspective is often missing, despite pronounced differences between men and women in longevity, dementia prevalence, and healthcare utilization.

This study addresses these gaps by identifying patterns in hospitalization risk among long-lived individuals aged 85 and above. We examine how hospitalization risk varies with age, comorbidity, long-term care dependency, nursing home residence, and proximity to death, and how these relationships differ by dementia status. Given the predominance of women among the long-lived, we also adopt a gender perspective.

We focused on long-lived individuals because dementia incidence and mortality rates are highest in this group (for Germany see [15]). Moreover, more than half of individuals already suffer from dementia by the time of death at age 85, with the prevalence continuing to increase with advancing age [16].

We took Germany as an example because it has one of the most rapidly ageing populations in the world [17], with long-lived individuals having doubled in recent decades, a growth far greater than that of older people aged 65 and over [18]. The ageing of the baby boomers in combination with the future gains in life expectancy will intensify this increase [18]. In Germany, all of the population is covered for a core set of health services which most importantly include outpatient treatment and hospital care, long-term care and living in a nursing home. Health care expenditures rise markedly with age and at the end of life, and the hospital sector constitutes the largest driver of costs in Germany’s health system. This reflects the country’s large hospital network, high number of hospital beds, and relatively long lengths of inpatient stay compared with other OECD countries [19]. The long-term care in Germany is financed mainly through the statutory long-term care insurance introduced in 1995 (Pflegeversicherung) and covers long-term care expenditures independent from whether people live in private homes or nursing homes. All relevant regulations on long-term care insurance can be found in Book XI of the German Social Code [20]. We hypothesized that having a dementia diagnosis, experiencing the end of life, being dependent on long-term care, living in a nursing home, and an increasing number of comorbidities increases the risk of hospitalization. Furthermore, a diagnosis of dementia in combination with the other factors generally increases the risk of hospitalization above and beyond the risk of the single factors. This increase is expected to be more pronounced in men than in women.

## Methods

### Data

The study analyzed a random sample of data from 250,000 individuals insured by the Allgemeine Ortskrankenkasse (AOK) who were born in 1954 or earlier. The sample was drawn in 2004, the first year in which health claims data became available for this public health insurer. AOK covers approximately one-third of the German population aged 50 and older and more than 50% of those aged 90 and above. The AOK granted access to the random sample, which represents approximately 2% of all AOK-insured individuals and includes both community-dwelling and nursing home residents. Our study had access to data on inpatient and outpatient treatment for these individuals, and we were able to follow them through 2019 thanks to a unique identifier. The AOK population has a lower socio-economic status compared to the general population, although this difference is more pronounced in younger people [21, 22].

We followed the 1918–1923 birth cohort (*n* = 4,065 men and 13,302 women) from age 85 onward (Table 1). Follow-up began when individuals reached age 85, meaning that entry into observation occurred between 2004 (for those born in 1918) and 2009 (for those born in 1923). By the final year of observation in 2019, individuals born in 1923 could reach a maximum age of 95, while those born in 1918 could reach up to age 101 (see Supplementary Material Table 1). To ensure comparability across the birth years, analyses were restricted to the age interval 85–95. Individuals who survived beyond age 95 (*n* = 321 men and 1,645 women) remained in the sample, but their observations after age 95 were excluded; thus, they were right-censored at age 95 [24, 25].

### Current Hospitalization

We used inpatient care data, which are measured by the day, to capture individuals who were hospitalized at least once in a specific quarter.

### Ever Hospitalization

We coded ever hospitalization an “ever variable” which had a value of one from the first quarter an individual had been hospitalized and zero otherwise.

### Explanatory Variables

Age, dementia status, quarter of death, comorbidity, nursing home residency, and long-term care were treated as time-varying variables, meaning that their values may change during the follow-up period. Age was measured in years and updated continuously from age 85 until death or the end of the observation period. The remaining variables were defined as “ever” variables, switching from zero to one at the time of their first occurrence and remaining one thereafter. A dementia diagnosis was defined by using the International Statistical Classification of Diseases and Related Health Problems, Tenth Revision, German Modification (ICD-10-GM 2024) [23] combined ICD codes F00-F03, F05.1, G30, G31.0. Because the data are available on a quarterly basis, diagnoses were assigned to the middle of the quarter, death is given by the exact month. We used an established validation procedure for dementia diagnoses to ensure accuracy and avoid overestimation (see Supplementary Material Table 2 [24, 25]. Dementia status takes the value one from the first validated diagnosis onward and zero beforehand, thereby distinguishing periods with dementia (PwD) from periods without dementia (non-PwD). This procedure produced age-specific prevalence estimates comparable to those reported in previous studies [24]. The variable death was operationalized by indicating whether individuals were observed in their quarter of death, switching from zero to one in that quarter. Comorbidities were treated as time-varying variables. We used ICD diagnoses from the Elixhauser comorbidity index [26] coding each diagnosis as one from the first time the diagnosis was observed and zero beforehand. All diagnoses were then combined into an unweighted summary score, which we divided into three terciles (Score < = 5; 5 < Score < = 8; Score > 8). Living in a nursing home was defined by switching from zero (living in private household) to one (living in a nursing home). Long-term care was defined as receiving benefits from the German long-term care system and was likewise coded as switching from zero to one from the first recorded receipt of benefits onward.

### Analysis Strategy and Statistical Analysis

Both in the univariate and multivariate analysis, we explored current hospitalization which is the occurrence of hospitalization in a specific quarter. In the multivariate analysis, including both fixed and random effects, we estimated a two-level mixed-effects linear probability model (Equ. 1) of being hospitalized ($\:{hosp}_{ij})$ or not in a specific quarter (j) with repeated measures on the individual level (i). The linear probability model allows coefficients to be interpreted directly as percentage-point changes in the probability of the outcome, facilitating interpretation and comparison across models.

In the base model, we included several fixed effects to capture systematic determinants of the outcome. Age was modeled using a quadratic polynomial (β_1_age_j_ + β_2_age_j_²) to allow for potential non-linear age trajectories. Additional fixed effects were included for quarter of death (β_3_death_j_), nursing home residence (β_4_nh_j_), long-term care status (β_5_ltc_j_), and comorbidity (β_6_comorb_j_), capturing factors that may influence the outcome at a given observation time. The parameter β_0_ represents the overall intercept, while β_1_–β_6_ denote the corresponding fixed-effect coefficients.

To account for repeated observations within individuals and unobserved heterogeneity, we specified individual-specific random effects. Each individual was allowed to have a random intercept (u_0_) and a random slope for the linear age term (u_1_age_j_), capturing differences in baseline levels and age-related trajectories across individuals. In addition, these individual trajectories were allowed to vary by dementia status through a gender-specific random effect (u_2_dem_j_). We coded dementia status dem_ij_ by gender (men PwD, men non-PwD, women PwD, women non-PwD). The residual term ε_j_ represents the normally distributed error term.

1 $$\eqalign{ & \>hos{p_{ij}} = \beta {\>_0} + \beta {\>_1}ag{e_{ij}} + \beta {\>_2}age_{ij}^2 \cr & + \beta {\>_3}deat{h_{ij}} + \beta {\>_4}n{h_{ij}} + \beta {\>_5}lt{c_{ij}} \cr & + \beta {\>_6}comor{b_{ij}} + {u_{i0}} + {u_{i1}}ag{e_{ij}} + {u_{i2}}de{m_{ij}} + \varepsilon {\>_{ij}} \cr} $$

In the full model we interacted the fixed effects with the gender-specific dementia status dem_ij_.

2 $$\eqalign{ & \>hos{p_{ij}} = \beta {\>_0} + \beta {\>_1}ag{e_{ij}}*de{m_{ij}} + \beta {\>_2}age_{ij}^2 \cr & + \beta {\>_3}deat{h_{ij}}*de{m_{ij}} + \beta {\>_4}n{h_{ij}}*de{m_{ij}} + \beta {\>_5}lt{c_{ij}}*de{m_{ij}} \cr & + \beta {\>_6}comor{b_{ij}}*de{m_{ij}} + {u_{i0}} + {u_{i1}}ag{e_{ij}} + {u_{i2}}de{m_{ij}} + \varepsilon {\>_{ij}} \cr} $$

For the base model we present the estimated coefficients which give the absolute increase in probability as compared to the reference group for categorical variables, and the absolute increase in probability with age increasing by one year for the linear term of the age polynomial. In the full model, we present the results as marginal effects and their 95% confidence intervals (95% CI). All analyses were performed using Stata 17.0.

## Results

### Descriptives

We observed 4,065 men (23%) and 13,302 women (77%) up to a maximum age of 95 (Table 1). Among men, average age for non-PwD was 87.89 years, and 88.79 years for PwD; among women it was 88.05 years (PwD) and 89.12 years (non-PwD). The percentage of current hospitalization was 15.3% among men, and 13.6% among women, with large differences between PwD and non-PwD (male PwD: + 9.8 PP, female PwD: +7 PP). Ever-hospitalization was around 90% for both gender, with PwD having higher percentages than non-PwD (male PwD: + 5.7 PP, female PwD: +4.6 PP).

Hospitalization at death was higher among men (62.35%) than women (55.21%), did not differ much by dementia status among men (-0.02 PP) and was lower for female PwD than for non-PwD (-6.54 PP). Less than half of the men (43.59%) lived in a nursing home, but more than half of the women (52.39%) did. Among men, 18.60% were in need of long-term care, among women the percentage was higher at 25.22%. PwD were more likely to live in a nursing home (men: +13.28 PP, women: +9.66 PP), to require long-term care (men: +16.32 PP, women: +21.43 PP), and to have higher numbers of comorbidities (men high: +5.88 PP, women high: +3.58 PP). At each age, PwD were more likely to be hospitalized than non-PwD, but the difference decreased with age (Supplemental Figure S1). In each group, the prevalence of hospitalization was lower for women than for men. For non-PwD, the prevalence increased with age, whereas for PwD it seemed to remain stable (men) or even decrease (women).

**Table 1:** Descriptives: in percentages

	Non-PwD	PwD	Total	*p*-value
	**Men**			
Current hospitalization	12.81	22.6	15.31	< 0.001
Ever-hospitalization	88.39	94.09	91.17	< 0.001
Hospitalization at death	62.36	62.34	62.35	0.991
Nursing home	37.12	50.4	43.59	< 0.001
Long-term care	10.65	26.97	18.6	< 0.001
Comorbidities low	35.44	30.3	32.94	< 0.001
Comorbidities medium	36.45	35.71	36.09	< 0.001
Comorbidities high	28.11	33.99	30.97	< 0.001
Average age	87.89	88.79	88.12	< 0.001
Total N	2,085	1,980	4,065	
	**Women**			
Current hospitalization	11.12	18.08	13.59	< 0.001
Ever-hospitalization	87.21	91.83	89.93	< 0.001
Hospitalization at death	59.14	52.6	55.21	< 0.001
Nursing home	46.7	56.36	52.39	< 0.001
Long-term care	12.6	34.03	25.22	< 0.001
Comorbidities low	35.91	32.09	33.66	< 0.001
Comorbidities medium	35.61	35.85	35.75	< 0.001
Comorbidities high	28.48	32.06	30.59	< 0.001
Average age	88.05	89.12	88.43	< 0.001
Total N	5,467	7,835	13,302	

### Multivariate Analysis of Current Hospitalization

In the base model (Table 2), women had a lower probability of hospitalization than men, and PwD a lower than non-PwD: compared to male non-PwD, the probability was + 0.10 (*p* < 0.001) higher for male PwD, -0.02 (*p* < 0.001) lower for female non-PwD, and + 0.06 (*p* < 0.001) higher for female PwD. There was no significant linear increase in probability with age, but the quadratic term showed a U-shaped increase of 0.02^E−100^ (*p* < 0.001) with age^2^. The quarter of death increased the probability by + 0.41 (*p* < 0.001). Living in a nursing home increased it by + 0.01 (*p* < 0.001) and being dependent on long-term care reduced it by -0.04 (*p* < 0.001). The probability increased with increasing number of comorbidities: +0.06 (p-value < 0.001) for a medium number of comorbidities and + 0.10 (p-value < 0.001) for a high number of comorbidities.

**Table 2:** Base model: Coefficients of mixed effects model (E^− 100^=0.01)

Variable	Coefficients	Standard Error	*p*-value
Dementia status by gender			
Men non-PwD (RG)			
Men PwD	0.1	0.0048	< 0.001
Women non-PwD	-0.02	0.0023	< 0.001
Women PwD	0.06	0.0028	< 0.001
Age*E^− 100^	-0.08	0.0008	0.29
Age*Age*E^− 100^	0.02	0.0001	< 0.001
Quarter of death			
no (RG)			
Yes	0.41	0.0028	< 0.001
Nursing home			
No (RG)			
Yes	0.01	0.0016	< 0.001
Long-term care			
No (RG)			
Yes	-0.04	0.0028	< 0.001
Comorbidity			
Low (RG)			
Medium	0.06	0.0015	< 0.001
High	0.1	0.0016	< 0.001
Const	0.07	0.0026	< 0.001

**Fig. 1:**
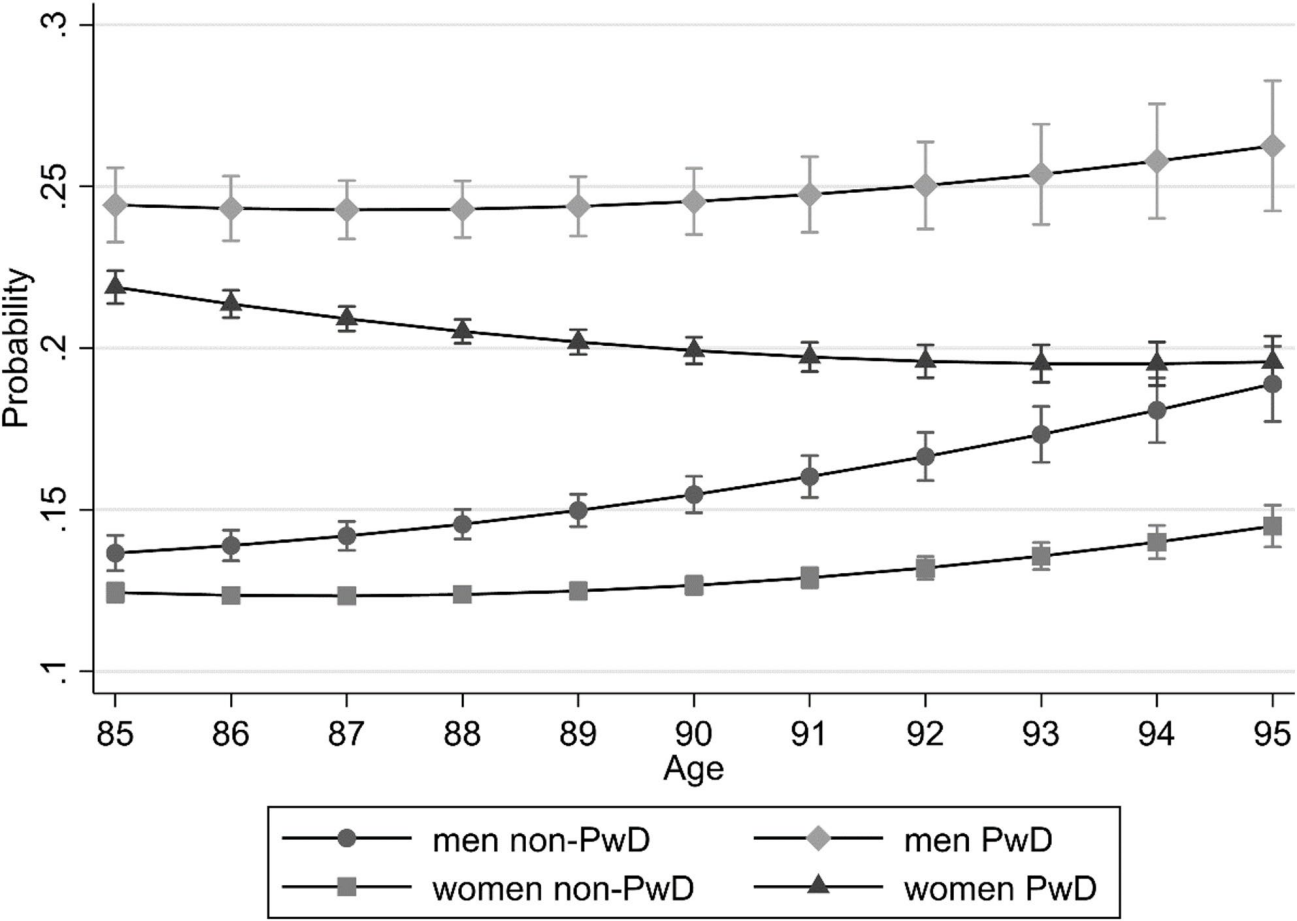
Age-specific probability of hospitalization by dementia status and gender. Predictive margins of the full regression model controlled for quarter of death, nursing home residency, long-term care, and comorbidity

In the full model including interaction effects, the main effects of gender and dementia remained unchanged. Men always had a higher probability of hospitalization than women, and PwD a higher than non-PwD. The probability of hospitalization (p_x_) at age x (Fig. 1) was always higher for PwD than for non-PwD and for males than for females. The increase of the probability with age was highest for male non-PwD (+ 0.052 from p_85_=0.14 [95%CI = 0.13–0.14] to p_95_=0.19 [95%CI = 0.18–0.20]), followed by male PwD (+ 0.018 from p_85_=0. 24 [95%CI = 0.23–0.26] to p_95_=0.26 [95%CI = 0.24–0.28]) and female non-PwD (+ 0.021 from p_85_=0.12 [95%CI = 0.12–0.13] to p_95_=0.14 [95%CI = 0.14–0.15]); but it was negative for female PwD (-0.023 from p_85_=0.22 [95%CI = 0.21–0.22] to p_95_=0.20 [95%CI = 0.19–0.20]), where ultimately the probability of male non-PwD was reached at the highest ages.

### Quarter of Death (Fig. 2A)

In non-death quarters men always had significantly higher probabilities of hospitalization than women, and PwD always had significantly higher probabilities than non-PwD. The difference (d) in the probability between PwD and non-PwD was higher for men (d = + 0.10; p_non−PwD_=0.13 [0.13–0.13], p_PwD_=0.23 [0.22–0.24]), than for women (d = + 0.08; p_non−PwD_=0.11 [0.11–0.11], p_PwD_=0.19 [0.19–0.20]). The quarter of death had the largest effect of all factors on hospitalization. However, this increase was modulated by gender and dementia status: in men the probability was independent of dementia status (d = 1; p_non−PwD_=0.61 [0.60–0.63], p_PwD_=0.62 [0.62–0.64]), in women the probability was lower than among men and lowest in those with dementia (d=-0.04; p_non−PwD_=0.58 [0.58–0.59], p_PwD_=0.54 [0.53–0.55]).

### Nursing Home (Fig. 2B)

Living in a nursing home only marginally changed the probabilities of hospitalization, however, these small changes depended on gender and dementia status. Among those living in private households, men had a higher probability of hospitalization than women (non-PwD: d = + 0.03; PwD: d = + 0.04), and PwD a higher probability than non-PwD (men: d = + 0.10; women d = + 0.09). Living in a nursing home led to a slight increase for female non-PwD (d = + 0.02: private household: *p* = 0.12 [0.12–0.12], nursing home *p* = 0.14 [0.14–0.14]), a slight decrease for female PwD (d=-0.01: private household: *p* = 0.21 [0.21–0.22], nursing home *p* = 0.20 [0.20–0.20]), and to unchanged probabilities for men regardless of the dementia status (d = 0.01 private household: *p* = 0.25 [0.24–0.26], nursing home *p* = 0.24 [0.22–0.25])).

### Long-Term Care (Fig. 2C)

Being dependent on long-term care had again a small but gender and dementia-specific effect on hospitalization. Among those not dependent on long-term care men had a higher probability of hospitalization than women (non-PwD: d = + 0.02, PwD: d = + 0.04), and PwD a higher probability than non-PwD (men: d = + 0.10, women: d = + 0.08). Being dependent on long-term care did not significantly change the probability for men, but significantly decreased it for women, particularly for those with dementia (non-PwD: d=-0.3; no long-term care: *p* = 0.13 [0.13–0.13], long-term care *p* = 0.10 [0.9 − 0.11]; PwD: d= -0.5; no long-term care: *p* = 0.21 [0.21–0.21], long-term care *p* = 0.16 [0.16–0.17]).

**Fig. 2:**
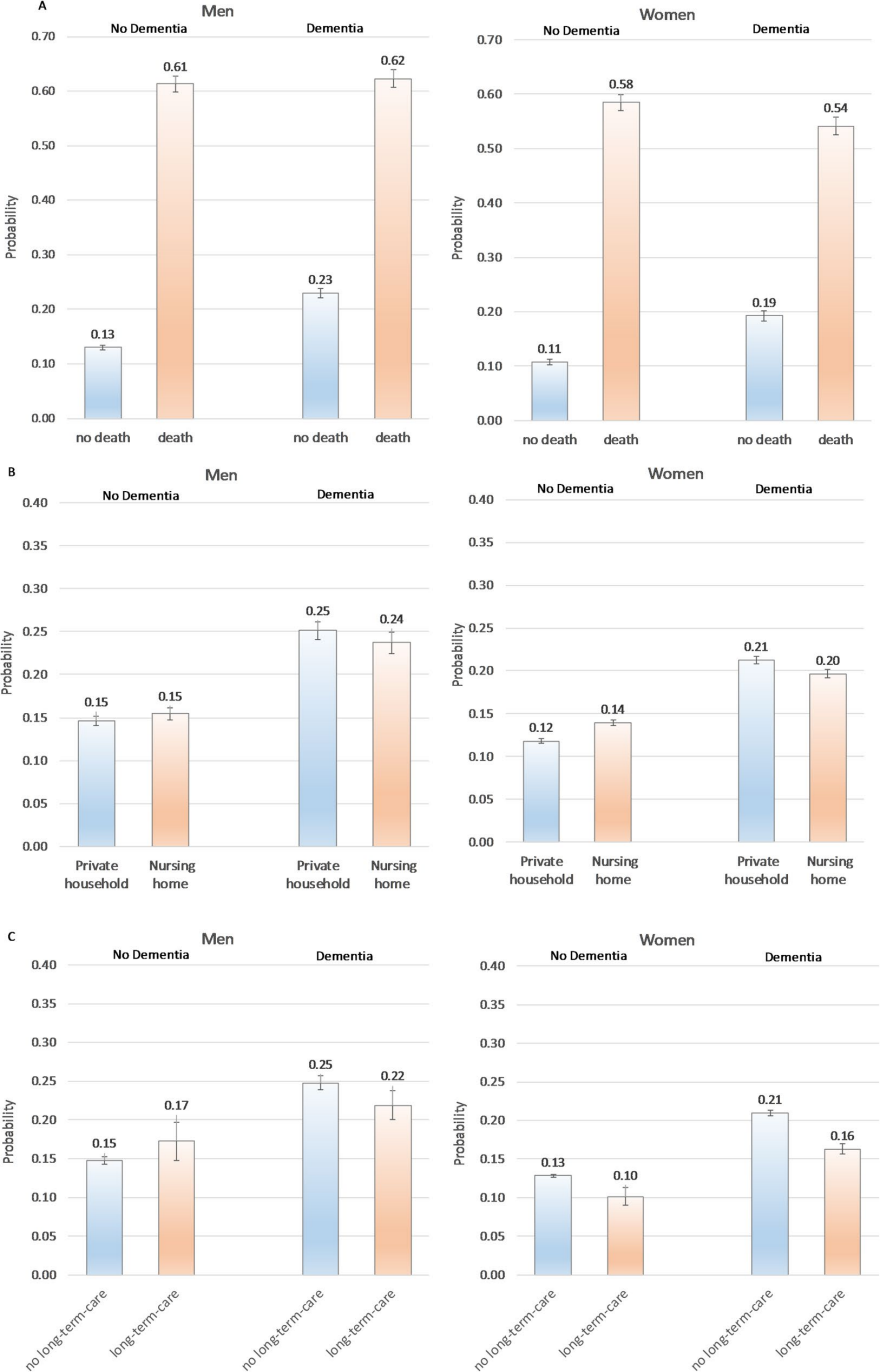
Probability of hospitalization by dementia status and gender for the quarter of death (**A**), nursing home residency (**B**), and being dependent on long-term care (**C**). Predictive margins of the full regression model

### Comorbidity (Fig. 3)

As the number of comorbidities increased, also the probability of hospitalization increased. Again, we find the pattern that men had a higher probability than women and PwD a higher than non-PwD, but there were no additional moderating effects

**Fig. 3:**
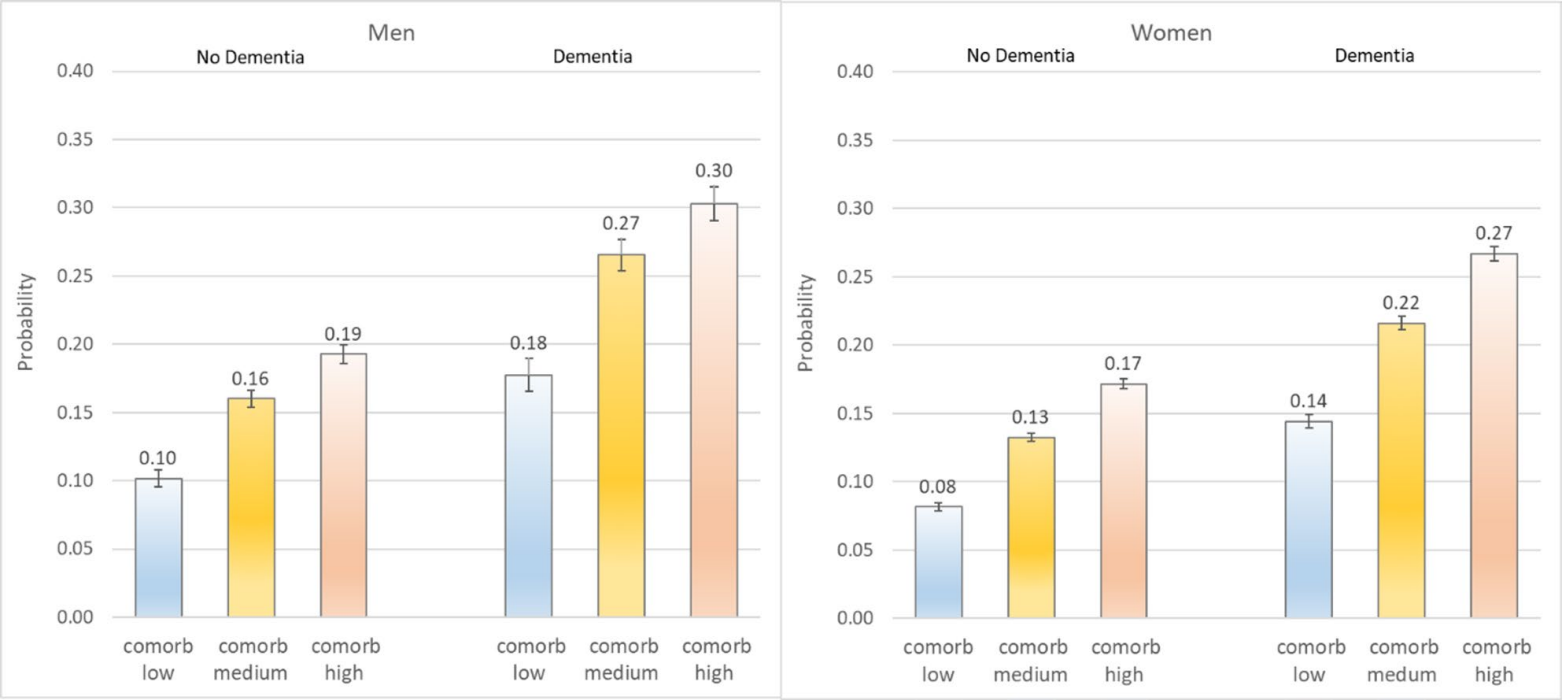
Probability of hospitalization by dementia status and gender for comorbidity levels. Predictive margins of the full regression model

## Discussion

This study examined hospitalization patterns among individuals born between 1918 and 1923, followed from ages 85 to 95. Our analysis indicated distinct gender and dementia-related differences in the probability of hospitalization, influenced by factors such as age, dementia status, end of life, comorbidities, and residential arrangements.

In our study, as expected, people with dementia had a higher probability of hospitalization in general. This finding is in line with a meta-study looking at hospital admissions in people with dementia compared to those without, which estimated a combined excess risk of 42% when controlling for age, sex, and physical comorbidities [27]. Dementia patients have consistently shown higher hospitalization rates than non-dementia patients in a previous study looking at ambulatory care-sensitive conditions, where proactive outpatient care could prevent the need for hospitalization [28]. This is despite that dementia may often be associated with worse outcomes from hospitalization, including worsening cognitive status. Reasons for higher hospitalization rates are likely multifactorial. Healthcare utilization tends to increase with the progression of dementia, particularly from the first to second year after diagnosis, partly because of emergency visits and hospitalization [29]. Underlying conditions that increase the risk of dementia (e.g. stroke) or develop in the setting of dementia (e.g. dysphagia, which increases the risk of pneumonia), the impaired ability to self-manage chronic conditions, recognize symptoms and alert others, may pose significant diagnostic and management challenges for primary care clinicians [30]. Furthermore, people with dementia may actually be sicker for similar levels of illness (e.g., more likely to experience delirium and functional decline due to acute illness [31]. In our study we controlled for the number of comorbidities, which therefore does not explain the increased probability of hospitalization for people with dementia. However, there is evidence from Finland that in the last two years of life, at all levels of comorbidity, people with dementia had lower hospitalization rates than those without [32]. Contrary to this study, our study does not only explore deceased individuals, but controls for the end-of-life effect. And as discussed below, we indeed find a lower hospitalization probability for women with dementia as compared to those without dementia, while for men the probability did not appear to depend on the presence of dementia.

Gender differences in hospitalization have been widely documented, with higher hospitalization rates among men reported across multiple care contexts, including nursing home [33], private households [34], and end-of-life care [35]. These findings are consistent with several biological and social interpretations discussed in the literature [34]. First, the presence of comorbidities is consistently associated with increased hospitalization risk among individuals with dementia (e.g. [27, 36]). Although men have a lower life expectancy than women and may benefit from a survivor advantage at advanced ages, whereby only the healthiest individuals remain, our findings suggest that men have more comorbidities than women. This pattern was independent from dementia status, and may reflect possible differences in disease progression or the accumulation of comorbidities [34, 37]. However, the present study does not permit direct examination of these mechanisms. Second, in our study women consistently demonstrated lower hospitalization probabilities than men across all comorbidity levels. Previous studies suggest that men with dementia may be more likely to undergo acute hospital care [35], whereas women with dementia appear to utilize more outpatient care without transfer to hospital until death [34]. This pattern may also help explain why the risk of hospitalization for women with dementia decreases with age.

Our analysis revealed four significant gender- and dementia-specific patterns reflecting the complex interplay between dementia status and hospitalization. First, in the quarter preceding death, women with dementia appeared to have a lower probability of hospitalization compared to their female counterparts without dementia, while men’s probability appeared unchanged by the presence of a dementia diagnosis. Previous research indicated hospitals as common places of death in Germany [38–40], followed by dying in one’s own home [40, 41]. Previous end-of-life studies have shown that among nursing home residents, those with dementia were less likely to be hospitalized than those without [14] and that men were more likely to be hospitalized than women [14, 42]. The avoidance of hospitalization at the end of life, e.g. by better geriatric training of physicians [43], may reduce the burden of treatment [44], and may help to improve quality of life, as well as prevent potentially inappropriate and overly aggressive treatment [45, 46]. But there is also evidence that patients dying with dementia may receive poorer quality end-of-life care than e.g. patients dying from cancer in several important areas of end-of-life care [47]. On the other hand, data from the Swedish Register of Palliative Care showed that death in hospitals may be associated with poorer quality of end-of-life care compared to death in nursing homes [35].

Second, women who were dependent on long-term care appeared to have a lower probability of hospitalization compared to their non-dependent counterparts, irrespective of dementia status. A similar pattern was observed among men with dementia, whereas among men without dementia, long-term care dependency did not appear to influence hospitalization risk. In Germany, albeit long-term care dependency is closely associated with nursing home residency, a substantial proportion of individuals requiring long-term care continue to live in private households. As a result, the effect of long-term care dependency is intertwined with residential status, as well as the distinct morbidity profiles of individuals in different living arrangements. These factors, in turn, may contribute to variations in hospitalization risk, as discussed in the following section.

Third, the probability of hospitalization associated with residing in a nursing home, compared to living in a private household, appeared to be notably higher only among women without dementia. Conversely, individuals with dementia exhibited no significant difference in hospitalization probability between nursing home residents and those living in private households, irrespective of gender. This observation contrasts with findings from a scoping review [48], which indicated substantially elevated hospitalization rates among German nursing home residents compared to non-institutionalized individuals. The discrepancy stems partly from our study’s control for multimorbidity, a factor identified as significantly higher among nursing home residents [33], who typically exhibit increased frailty and comorbid conditions compared to their counterparts in private households. The hospitalization rate has been reported to be particularly elevated during the initial three months following nursing home admission, commonly associated with underlying health conditions such as multimorbidity, chronic obstructive pulmonary disease (COPD), heart failure, cancer, and dementia. Frequently reported causes of hospitalization among nursing home residents include also falls, fractures, and cardiovascular or respiratory complications [48]. In a systematic review [14] the authors highlighted two older studies examining hospitalization risks between nursing home residents and private household residents. The first [49] reported higher hospitalization rates among nursing home residents for femur fractures and pneumonia, whereas individuals in private households were more frequently hospitalized due to heart disease. The second [50], controlling for age, sex, and mortality, found reduced hospitalization rates among individuals who resided in nursing homes for over a year compared to their counterparts living in private households. This review concluded that, toward the end of life, nursing home residents with dementia experienced lower hospitalization rates than those without dementia. Echoing our results, it emphasized that gender and age factors might differently influence hospitalization risks when comparing dementia residents to those without dementia. Additionally, managing dementia patients is widely recognized as challenging, particularly in private home settings, which may contribute to hospitalizations for conditions that could otherwise be managed outside hospital environments [5].

Fourth, age profiles of hospitalization probabilities tended to differ by gender and dementia status. While women with dementia exhibited the second highest hospitalization probability—surpassed only by men with dementia—it declined with increasing age, while all others increased. Contrary to our initial hypothesis, among men, it was those without dementia who appeared to have the greatest increase in hospitalization probabilities with age, followed by men with dementia. Although these gender- and dementia-specific variations were relatively small compared to the overall effect size of dementia, they nonetheless suggest a nuanced interaction between gender, age, dementia status, and hospitalization risk. A systematic review [33] concluded that the influence of age on hospitalization in nursing home populations was less clear than the gender effect with some studies showing increasing rates of admissions with increasing age while others found decreasing hospitalizations above approximately 80 to 85 years, particularly for women in Germany [51].This contrasts the general assumption that health care utilization increases with older age.

The observed hospitalization patterns described above are likely shaped by the German healthcare system in several ways. Universal coverage and a strong hospital sector with high bed capacity and long lengths of stay may lower thresholds for admission and increase overall hospitalization rates. This may be especially relevant for men, people with dementia, and those with multiple comorbidities. At the same time, the availability of statutory long-term care insurance supports care in community and nursing home settings, which may reduce hospital transfers for certain groups, especially older women with dementia. This may explain the observed decline in hospitalization with age in this subgroup. The pronounced increase in hospitalizations near the end of life is consistent with both higher care needs and a hospital-oriented system. The modestly higher rates among nursing home residents likely reflect a balance between continuity of care in these settings and limited on-site medical resources, which can necessitate hospital transfers. Overall, these findings suggest that hospitalization patterns are driven not only by clinical factors but also by system-level characteristics of the German healthcare system.

### Strength

This study has a number of strengths. The data used are provided by Germany’s largest health insurance provider (AOK) with a high number of individuals also at the highest ages. Also, our sample includes the complete population living in the community, both in private households and institutions. To increase the validity of the diagnoses, we reduced the number of false-positive diagnoses by applying an internal validation procedure. In contrast to data collected from healthcare providers directly, health claims data are independent of the choice of the physician, hospital, and place of residence. In contrast to self-reported disease, hospital stays are not affected by recall bias. The bias due to self-selected dropouts is minimal compared to the bias in survey-based studies, because the data are collected irrespective of whether patients move or change health providers. This is particularly important, as evidenced by the high incidence of dementia among those who move [52].

### Limitations

Despite these many strengths there are also a number of limitations. We used ICD-10 codes that were officially reported to the health insurance company by physicians. We have no information on the accuracy and appropriateness of the diagnosis measurement, definition, and reporting quality used by physicians and cannot validate these diagnoses externally. We do not differentiate dementia by etiology because approximately 45% of diagnoses are categorized as unspecified dementia (F03), and there is no information available on dementia severity. Additionally, this study lacks information on medication use. These data limitations may affect the interpretation of hospitalization patterns. Not differentiating dementia by etiology combines clinically distinct conditions with different progression and comorbidity profiles, which may mask variation in hospitalization risk. The lack of information on dementia severity further limits interpretation, because hospitalization risk may differ substantially between early and advanced stages of the disease. In addition, missing data on medication use may bias the results because certain drugs can increase the risk of complications that lead to hospitalization [53, 54]. Furthermore, although our diagnosis variables are time-varying and may change for individuals over time, the exact date of diagnosis is not available. Therefore, diagnoses were assigned to the midpoint of the respective quarter. However, in previous analyses, we compared our estimates of dementia incidence and prevalence with results from international epidemiological studies and found that the age-specific profiles fit well [24, 55]. In addition, nursing home residence and long-term care were coded as “ever” variables, indicating whether an individual had experienced these states at any point during the observation period. While this approach may, in principle, introduce misclassification by not capturing the exact timing or potential transitions, such bias is likely limited in the German context. Transitions back from nursing homes to community settings are relatively uncommon, and long-term care dependency typically reflects a sustained need rather than a temporary state. Thus, the “ever” coding primarily captures a persistent exposure rather than a reversible condition. Nevertheless, this approach does not account for the timing or duration of care, and future studies with time-varying measures could provide a more precise assessment of how changes in care status influence hospitalization risk. Finally, the proportion of people with a low socioeconomic status is higher among AOK members than among members of other statutory health insurance companies, as well as among private health insurance company members [21]. This difference can be explained partly by the older age structure of the AOK population compared to the total German population. This may limit the generalizability of the findings, as lower socioeconomic status is associated with poorer health, higher comorbidity burden, and potentially higher hospitalization rates. Consequently, the absolute levels of hospitalization observed in this study may be overestimated compared to the overall German population. However, relative differences between groups (e.g., by sex, dementia status, or care dependency) are likely less affected. Prior evidence suggests that socioeconomic differences between AOK insured individuals and the general population are more pronounced at younger ages and diminish in older age groups [21, 22], and their mortality rate is similar to the total German population [22]. Therefore, the findings for individuals aged 85–95 may be more representative of the total population. Further research on the total population covered by statutory health insurance would help to clarify the extent of a potential selection bias.

## Conclusion

Our analysis revealed significant gender disparities in hospitalization patterns among individuals aged 85 to 95. Men, especially those with dementia, have a higher probability of being hospitalized than women. This can further increase the substantial clinical and caregiving burdens associated with dementia for patients, families, and healthcare providers. In contrast, older women with dementia, especially at advanced ages and near the end of life, have the lowest hospitalization rates. While lower hospitalization rates may reflect a preference to avoid burdensome interventions, they also raise concerns that some women with advanced dementia may have limited access to necessary acute or end-of-life medical care. Individuals with dementia living in private households require particular attention because care arrangements that rely primarily on family caregivers and ambulatory services may struggle to manage their complex health needs. This increases their vulnerability to acute deterioration and hospitalization.

From a health policy perspective, these results suggest that health systems must adapt to accommodate the growing population of individuals aged 85 and older with complex care needs. Taken together, our findings underscore the importance of adopting gender-sensitive and context-specific approaches to geriatric care. They highlight the need for better outpatient management of acute and chronic conditions, stronger coordination between primary care providers, community services, and long-term care providers, and earlier advance care planning including palliative care.

## Supplementary Information

Below is the link to the electronic supplementary material.

Supplementary Material 1

## Data Availability

The data supporting the findings of this study are available on request from WIdO (webpage: https://www.wido.de/, mail: E-mail: wido@wido.bv.aok.de). The data are not publicly available due to privacy or ethical restrictions.
