# Supplementary material for: Modification of Hospitalization Risk by Gender and Dementia Status Between the Ages of 85 and 95 in a German Cohort Based on Health Claims Data
Source: BMC Health Serv Res. 2026 May 8;26:670. doi: 10.1186/s12913-026-14575-2 (PMC13157667; doi:10.1186/s12913-026-14575-2)
Supplement: Supplementary file 1 — Supplementary Material 1 [file 12913_2026_14575_MOESM1_ESM.docx]

Supplemental Table 1: Schematic cohort chart of age by observation year and birth year

|  | Birth Year |  |  |  |  |
| --- | --- | --- | --- | --- | --- |
| Observation Year | 1919 | 1920 | 1921 | 1922 | 1923 |
| 2004 | 85 | ~~84~~ | ~~83~~ | ~~82~~ | ~~81~~ |
| 2005 | 85/86 | 85 | ~~84~~ | ~~83~~ | ~~82~~ |
| 2006 | 86/87 | 85/86 | 85 | ~~84~~ | ~~83~~ |
| 2007 | 87/88 | 86/87 | 85/86 | 85 | ~~84~~ |
| 2008 | 88/89 | 87/88 | 86/87 | 85/86 | 85 |
| 2009 | 89/90 | 88/89 | 87/88 | 86/87 | 85/86 |
| 2010 | 90/91 | 89/90 | 88/89 | 87/88 | 86/87 |
| 2011 | 91/92 | 90/91 | 89/90 | 88/89 | 87/88 |
| 2012 | 92/93 | 91/92 | 90/91 | 89/90 | 88/89 |
| 2013 | 93/94 | 92/93 | 91/92 | 90/91 | 89/90 |
| 2014 | 94/95 | 93/94 | 92/93 | 91/92 | 90/91 |
| 2015 | 95~~/96~~ | 94/95 | 93/94 | 92/93 | 91/92 |
| 2016 | ~~97~~ | 95~~/96~~ | 94/95 | 93/94 | 92/93 |
| 2017 | ~~98~~ | ~~97~~ | 95~~/96~~ | 94/95 | 93/94 |
| 2018 | ~~99~~ | ~~98~~ | ~~97~~ | 95~~/96~~ | 94/95 |
| 2019 | ~~100~~ | ~~99~~ | ~~98~~ | ~~97~~ | 95~~/96~~ |

Deleted ages are excluded from the analysis.

| 1. considering only verified diagnoses from the outpatient sector and discharge or secondary diagnoses from the inpatient sector. |
| --- |
| 1. The validation procedure also required that at least two types of physicians (general practitioners, neurologists, psychiatrists, or other specialists) had made a diagnosis of dementia in the same quarter, or that a diagnosis occurred simultaneously in both the inpatient and outpatient sectors. |
| 1. Additionally, dementia diagnoses were confirmed by occurrence in multiple consecutive years of data. |
| 1. If an individual died during the quarter in which their first dementia diagnosis was made, that diagnosis was still considered to be valid. |

Supplemental Table 2: Validation procedure of dementia diagnosis in German health claims data

Supplemental Figure 1: Prevalence of current hospitalization by age and dementia status


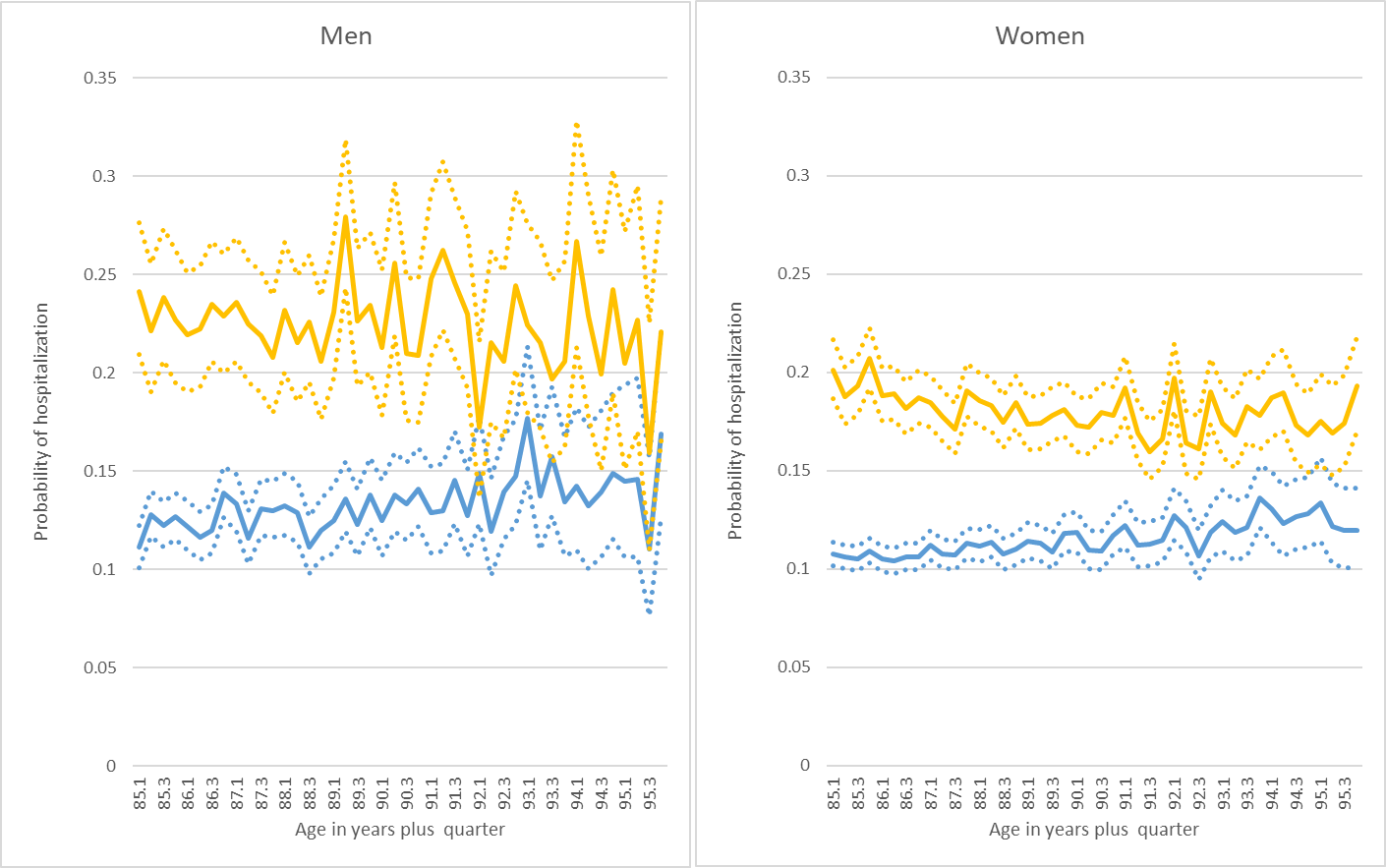


yellow solid line: with dementia, blue sold line: without dementia, dashed lines: 95% confidence intervals
